# Lymphomatoid granulomatosis with the central nervous system involvement as the main manifestation: a case report

**DOI:** 10.1186/s12883-023-03257-9

**Published:** 2023-05-29

**Authors:** Dawei Chen, Jing Zhou, Weiwen Lu, Liuzhe Lu, Bo Chen, Wenzhong Li

**Affiliations:** Department of Neurosurgery, The First Hospital of Jilin University, Changchun, Jilin Province China

**Keywords:** Lymphomatoid granulomatosis, Central nervous system, Immunohistochemistry, IFN-α

## Abstract

**Background:**

Lymphomatoid granulomatosis (LyG) is a rare extralymphatic lymphoproliferative disease characterized by lymphocytic invasion into vascular walls and damage to blood vessels. The lungs are affected in 90% of LyG cases, followed by the skin, central nervous system (CNS), kidneys and liver.

**Case presentation:**

Here we report a case of a young woman with LyG, with CNS involvement as the initial clinical manifestation. Computer tomography (CT) scans showed multiple nodular, patchy and flocculent high-density shadows in both lungs without mediastinal lymph node enlargement. Magnetic resonance imaging (MRI) scans showed multiple abnormal signal intensities in the right cerebellar hemisphere, frontal, parietal and temporal lobes, and dorsal brainstem, which became patchy and annular after enhancement. The post-operative pathological analysis of lesion samples confirmed the diagnosis of grade II LyG.

**Conclusions:**

LyG should be concerned in young adults showing multiple radiological brain and lung lesions. Resection and postoperative medication of steroid hormones and IFN-α may be effective in the treatment of LyG.

## Background

Lymphomatoid granulomatosis (LyG), initially reported by Leibow et al. [1], is closely associated with the Epstein-Barr virus (EBV) infection and the immunosuppression caused byhuman immunodeficiency virus (HIV) infection, T cell deficiency, use of immunosuppressive agents, etc [2, 3]. CNS involvement usually appears as a simultaneous or secondary symptomof pulmonary lymphomatoid granulomatosis (PLG) [4]. Primary lymphomatoid granulomatosis with the involvement of the central nervous system (CNS-LyG) is rare, and its association with EBV infection and cell phenotypes has not been fully elucidated [5, 6]. CNS-LyG is defined as a lymphoproliferative disease characterized by T-cell infiltration and B-cell proliferation of an indeterminate malignancy, according to the WHO guidelines in 2001 [7]. *The WHO Classification of Tumors of Hematopoietic and Lymphoid Tissues in 2008* proposed that LyG is a subtype of diffuse large B-cell lymphoma (DLBL) that can be histologically classified into the low-level (grade I-II) and high-level (grade III) [8]. Active treatment of the primary disease and eradication of the cause are given to LyG patients with a clear etiology or high-risk factors, and those without clear causes are treated based on pathological classification [9].

## Case presentation

A 23-year-old woman presented with hypomnesis and unsteady gait for 1 month. The patient presented with sore throat, fatigue, nasal obstruction after hard work at 10 days before admission. Oral antiviral and antibiotic drugs did not relieve the symptoms. Thereafter, progressive memory loss appeared, and the patients fell to the right when ready to walk. She had no fever, cough, mental disorders, and abnormal behaviors during the course of the disease. Abnormal previous history, medical history and family history were denied. Physical examinations showed a clear mind and normal communication. Rashes, erythema nodosum and enlargement of superficial lymph nodes were not found. Memory loss (forgetting to eat breakfast or the parking position of her car) was noticed, with cranial nerves (-). Horizontal nystagmus in the right eye was detected. The finger-to-nose test on the right and heel-knee-tibia test were poor. The Romberg sign was positive.

**Fig. 1:**
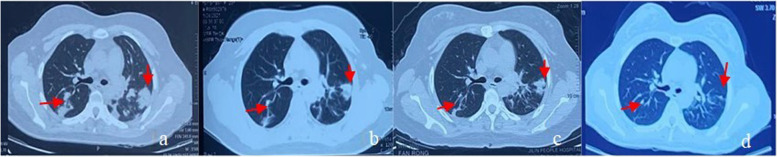
Lung CT scans on admission (**a**) showing multiple nodular, patchy and flocculent high-density shadows in both lungs without mediastinal lymph node enlargement, and those at 3 months (**b**), 6 months (**c**) and 18 months (**d**) after treatment

**Fig. 2:**
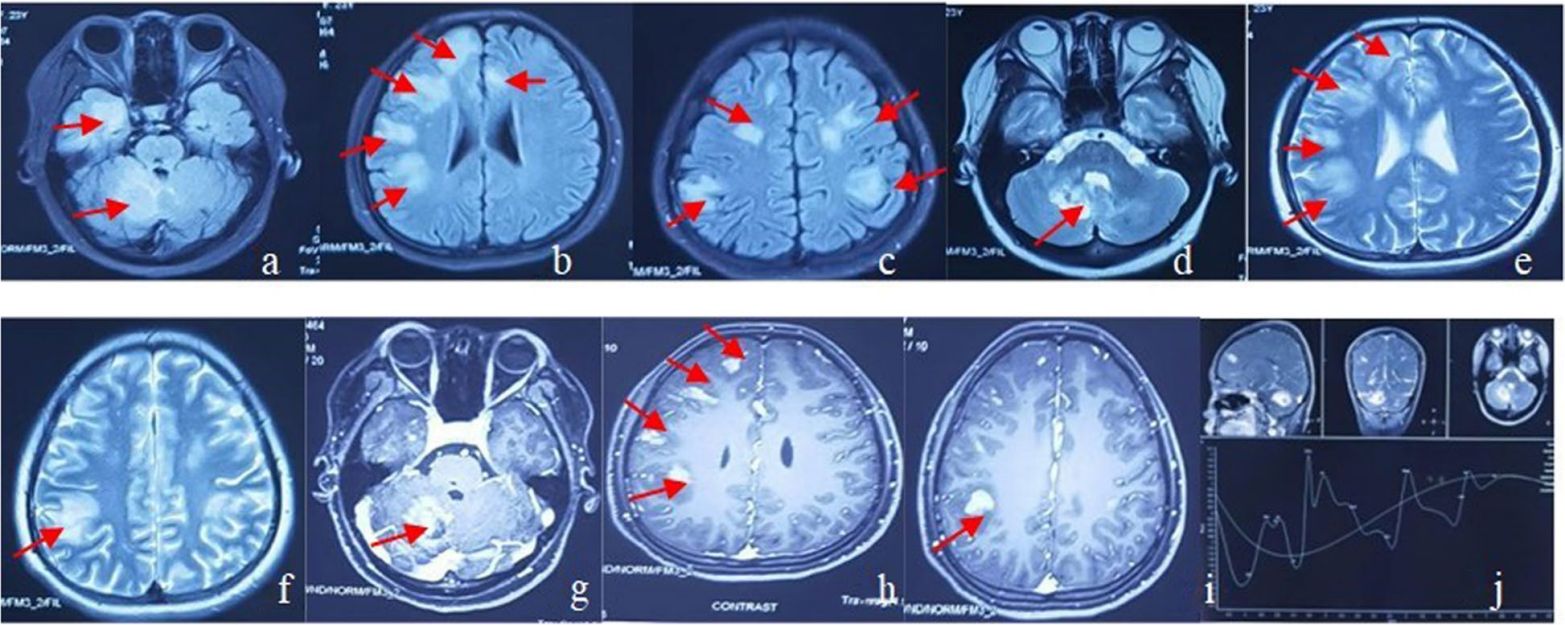
MRI scans showed multiple abnormal signal intensities in the right cerebellar hemisphere, frontal, parietal and temporal lobes, and dorsal brainstem, presenting an isointensity or high signal intensity on T1-weighed images (**a**-**c**), and a slightly high or high signal intensity on T2-weighed images (**d**-**f**). Patchy and annular lesions were visualized on contrast-enhanced MRI scans (**g**-**i**). ^1^H-MRS showed a slight increase in Cho and a slight decrease in NAA of the right cerebellar and left frontal lobe lesions, as well as a tall and broad Lip peak (**j**)

Laboratory testing showed that C-reactive protein (CRP), procalcitonin (PCT), erythrocyte sedimentation rate (ESR) and female tumor markers were normal. Serum (1,3)-β-D glucan (G-test), galactomannan (GM-test), purified protein derivative (PPD) skin test, HIV, treponema pallidum antibody (TP-Ab), antinuclear antibody (ANA) profile, antineutrophil cytoplasmic antibodies (ANCA) and neuronal nuclear antibodies (Hu and Ri) were negative. Bronchoscope findings were normal. Tumor cells were absent in the bronchoalveolar lavage fluid. The metagenomic next-generation sequence (mNGS) data showed that bacteria, fungi, parasites, Rickettsia/Mycoplasma/Chlamydia and *Mycobacterium tuberculosis* complex were negative. Six EBV sequences were detected. Lung CT scans showed multiple nodular, patchy and flocculent high-density shadows (0.3–3.1 cm) in both lungs without mediastinal lymph node enlargement (Fig. 1a). MRI scans showed multiple abnormal signal intensities in the right cerebellar hemisphere, frontal, parietal and temporal lobes, and dorsal brainstem, presenting isointensity or high signal intensity on T1-weighed images, and slightly high or high signal intensity on T2-weighed images (Fig. 2a-f). Patchy and annular lesions were visualized on contrast-enhanced MRI scans. A lesion sized 3.25 cm×3.41 cm×2.18 cm was found in the right cerebellum, accompanied by mild edema in the surrounding tissues (Fig. 2g-i). Proton magnetic resonance spectroscopy (^1^ H-MRS) showed a slight increase in choline (Cho) and a slight decrease in N-acetyl aspartate (NAA) of the right cerebellar and left frontal lobe lesions, as well as a tall and broad lipids (Lip) resonance peak (Fig. 2j). Video electroencephalograph (EEG) records were normal. After 3 days of intracranial decompression without steroid hormones, the opening pressure at the lumbar puncture was 160 mmH_2_O (1 mmH_2_O = 0.098 kPa). The cerebrospinal fluid (CSF) was colorless and transparent, and the white cell counts, sugar and protein levels in the routine testing of CSF were normal. Tumor cells were absent in the CSF. India ink staining, Cysticercosis antibody, the MycoDot test, the FTA-ABS test, aquaporin-4 (AQP4-IgG), myelin oligodendrocyte glycoprotein (MOG) antibody and myelin basic protein (MBP) antibody in the CSF were negative. The CSF IgG index was 0.57, and IgG oligoclonal bands (OCBs) were negative. In the Reiber’s diagram, IgG, IgA and IgM were all located in the R2, representing abnormal blood brain barrier (BBB) function without local synthesis of IgG. An initial diagnosis of intracerebral and pulmonary multiple lesions was considered, such as immune-mediated demyelinating disorders, Erdheim-Chester disease or primary central nervous system lymphoma.

**Fig. 3:**
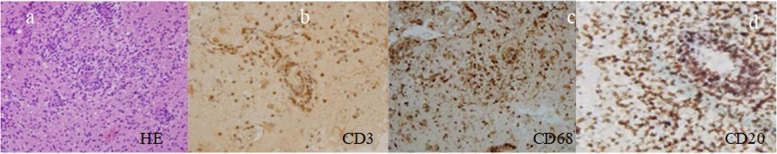
Postoperative pathological findings. H&E staining showed T-cell infiltration surrounding the blood vessel, perivascular lymphoid sheaths, damages of partial blood vessel walls and proliferation of vascular endothelial cells **a**. Immunohistochemical staining of CD3^+^ in heterocysts (**b**, magnification = ×200), CD68^+^ in infiltrated macrophages (**c**, magnification = ×200) and CD20^+^ in cells surrounding the blood vessel (**d**, magnification = ×400)

After 6 days of nourishing the nerves, lowering the cranial pressure, and antiviral treatment, the symptoms did not subside. Then, after communication, the patient was surgically treated with the resection of right cerebellar lesions at the 6th day after admission. Postoperative pathology showed T-cell infiltration surrounding the blood vessel, perivascular lymphoid sheaths, damageto partial blood vessel walls, and proliferation of vascular endothelial cells (Fig. 3a). Immunohistochemical staining showed: P53 (< 3%), IDH1 (-), NeuN (+), MBP (-), CD45RO (+), EGFR (-), CD79α (+), Ki-67 > 5%, GFAP (+), MAP-2 (sporadic+), EBER (-), CD3^+^ (Fig. 3b), CD68^+^ (Fig. 3c) and CD20^+^ (Fig. 3d). Gene rearrangements of T-cell receptor, immunoglobulin heavy chain, Kappa light chain and Gamma light chain were negative. Based on the laboratory and pathological findings, the patient was diagnosed as grade II LyG.

**Fig. 4:**
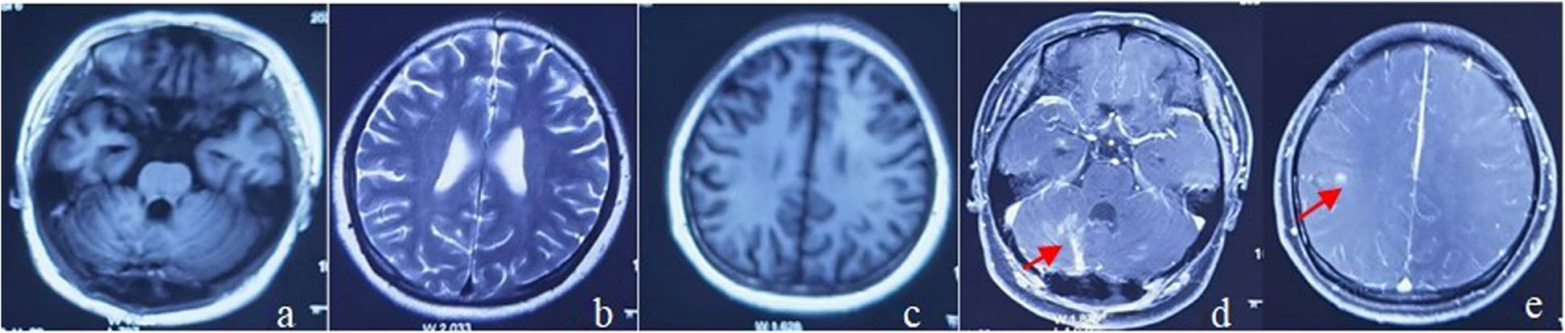
Basically subsided lesions after 3 months of INF-α treatment

**Fig. 5:**
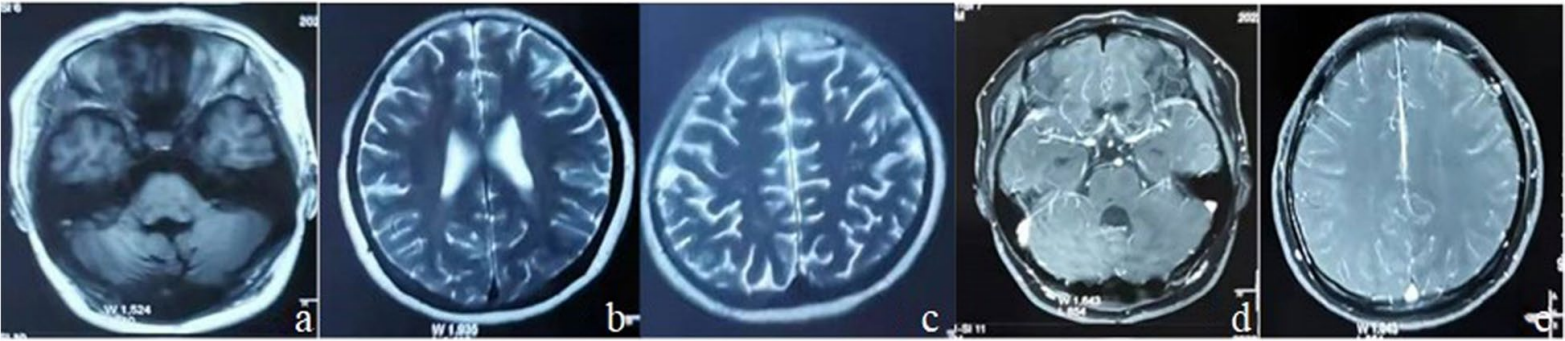
Completely subsided lesions after 6 months of INF-α treatment

**Fig. 6:**
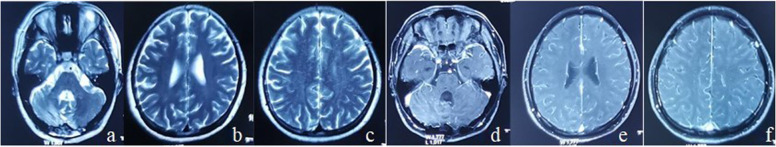
No new lesions detected at 18 months of follow-up

After obtaining informed consent to participate in further clinical investigation, the patient was subcutaneously administrated with prednisolone (PSL) at 60 mg/day and 600 mIU IFNα-2b, three times per week. Lesions in both lungs were absorbed (Fig. 1b), and multiple lesions and edema in the brain subsided basically (Fig. 4a-e) with gradually reduced dosage of PSL, until withdrawal at 3 months postoperatively. The patient presented a mild moon-face and normal findings of routine blood test. The dosage of IFNα-2b was adjusted to 750mIU, three times per week, but readjusted to the initial dosage due to the fever, and joint and muscle pain one month later. At six months postoperatively, the lesions in both lungs were further absorbed (Fig. 1c) and intracranial lesions subsided completely (Fig. 5a-e), thus achieving the withdrawal of IFN-α. Absorption of lesions in both lungs (Fig. 1d) was detected at 18 months of follow-up, and no new intracranial lesions formed up (Fig. 6a-f).

## Discussion and conclusions

LyG, as a systemic disease, mainly sets on at 40–60 years, and males are more susceptible than females (a male-to-female ratio ≥ 2:1). Its clinical manifestations vary substantially. It is reported that 67% of PLG patients exhibit clinical symptoms and signs of cough, dyspnea, and chest pain [10]. Typical features on chest CT images include single or multiple nodules of varying sizes or diffuse infiltrative lesions in the periphery of both lungs, which are distributed alongside the bronchovascular bundles or randomly distributed. Cavitation and pleural effusion can be detected. Enlargement of the hilar and mediastinal lymph node is usually non-detectable [11, 12]. Patients with CNS-LyG may have clinical symptoms of diplopia, mental disorder, ataxia, and epileptic seizures according to the location and extent of the lesions, and a small number of them may be asymptomatic [13]. Multiple, sporadic, punctate infiltrative lesions and mass-like lesions are visualized on MRI scans of patients with CNS-LyG. A space-occupying effect with surrounding edema is obvious in mass-like lesions [14–16]. On contrast-enhanced CT scans of patients with CNS-LyG, multiple punctate, linear, patchy, nodular and mass-like enhancing lesions can be observed, which may also evolve into irregular annular lesions. Gaha et al. [17] considered that multiple punctate or linear enhancing lesions are specific imaging manifestations of CNS-LyG. Lucantoni et al. [18] reported that the incidence of mass-like lesions in patients with primary LyG is much higher than that in patients with CNS-LyG (63.6% vs. 16.0%). ^1^ H-MRS contributes to the differential diagnosis of primary lymphomain the CNS, presenting an elevated Lip peak [19]. The involvement and extent of LyG lesions can be identified by positron emission tomography (PET)/CT, and an increased standard uptake value (SUV) [20].

The present case described a young woman with a normal immune function and without high-risk factors. Although lung CT scans showed pneumonia-like, mass-like, and nodular lesions, she did not present with respiratory symptoms. Her brain MRI findings were consistent with those previously reported [14]. A classic pathological triad of LyG was detected, including the polymorphous lymphoid cell infiltration, lymphocytic invasion of vascular walls, and granulomatous lesions. Epstein-Barr encoding region (EBER) in situ hybridization for EBV was negative. Based on laboratory testing, imaging findings and postoperative pathology, chronic inflammatory demyelinating polyradiculoneuropathy (CIDP), primary central nervous system lymphoma (PCNSL), Wegener’s granulomatosis, CNS vasculitis and Langerhans cell histiocytosis (LCH) could be excluded. In children, LyG demonstrates different features, like lower incidences of general discomfort, weight loss, hepatosplenomegaly and lymphadenopathy, as well as higher incidences of nervous diseases. However, both children and adults of LyG present similar chest X-ray and CT images, and brain MRI showing multifocal lesions in the white matter and high T2 signal in the grey matter surrounded by edema and sometimes with enhancement. The mean duration from the onset to the diagnosis of LyG is 8.5 months. LyG should be considered in those with typical imaging findings that cannot be speculated as other diseases. A timely diagnosis by surgery and postoperative pathology is needed to avoid misdiagnosis and poor prognosis.

Recognized guidelines are scant for the treatment of LyG. Therapeutic strategies for LyG include observation, surgery, steroid hormones, IFN and intravenous immunoglobulin (IVIG), rituximab, chemoradiotherapy, stem cell transplantation, bone marrow transplantation, and adoptive cellular immunotherapy for EBV [10, 21]. LyG patients are usually treated based on the histological grade [4, 9]. In a small proportion of patients, LyG gets stableorsubsides after the immune status is enhanced [22]. Patients with limited LyG lesions can be actively managed by surgery and postoperative systemic therapy. Glucocorticoids alone and their combination with immunosuppressive agents should be given to grade I and II LyG patients, respectively. Grade I and II LyG caused by immunosuppressive agents are treated with IFN and rituximab. Aggressive grade I and II LyG are treated with CHOP chemotherapy; while grade III is treated with R-CHOP chemotherapy or R-CVP. Non-responders can be subjected to stem cell or bone marrow transplantation [1, 21, 23]. The National Cancer Institute (NCI) guidelines recommends the use of IFN-α for low-grade LyG, the optimal dose of which is adjusted according to the efficacy and tolerance and lasts for 1–2 years. It is reported that the complete remission rate and progression-free survival of IFN-α-treated patients with low-grade LyG are 60% and 56%, respectively [24]. Through literature review, we propose the differential diagnosis of multisystem organ diseases with multi-invasive lesions in the CNS (Table 1).

**Table 1:** Differential diagnosis of multisystem organ diseases with the manifestations of multi-invasive lesions in the CNS.

Disease	Systemic manifestations	Radiological features	Histopathology	Treatment
CNS-LyG	CNS and systemic involvement (i.e., lungs, skin, etc.), respiratory and focal neurological deficits.	Diffuse infiltrative lesions and mass-like lesions in the brain. Linear, patchy, nodular, mass-like, and irregular ring-like lesions on contrast-enhanced CT scans.	Polymorphous lymphoid cell infiltration, lymphocytic vasculitis, lymphocytic invasion of vascular walls, and granulomatous lesions. CD68 (+), CD3 (+), CD20 (+).	Surgery, observation, steroid hormones, interferon, chemotherapy, radiotherapy, rituximab, stem cell transplantation and bone marrow transplantation.
CNS-ECD	CNS and systemic involvement (i.e., lungs, bone, etc.), bone pain, diabetes insipidus and cerebellar ataxia.	Occupying lesions or massive nodules in the brain, involving the dura and showing meningioma-like masses with obvious homogeneous enhancement.	Observation of lipid-rich foam cells or eosinophilic cytoplasmic histiocytes and fibroblasts. CD68 (+), CD1a (-), S-100 (+/-) and Birbeck granules (-).	Surgery, observation, steroid hormones, interferon, cytotoxic drugs, radiotherapy and targeted drugs.
CNS-T-LYP	CNS and systemic involvement (i.e., lungs, joints, etc.).	Multiple occupying and diffuse lesions in the brain with punctate, mass-like, and ring-like enhancement; peripheral edema; involvement of both brainstem and cerebellum.	Diffuse infiltration of massive microlymphoidocytes, perivascular lymphoid sheaths, abundant reticular fibers, no obvious mitotic phase. CD3 (+), CD4 (+) and CD8 (+/-).	Surgery, steroid hormones and immunosuppressants.
CNS-IgG4-RD	CNS and systemic involvement (i.e., pancreas, salivary lacrimal glands, kidneys, lungs, etc.).	Orbital pseudotumor, hypophysitis, diffuse thickening or masses in the endocranium/dura mater.	Massive lymphocyte and plasma cell infiltration with fibrosis, IgG-positive plasma cells > 40%.	Steroid hormones, rituximab, azathioprine and other immunosuppressants, imatinib and tocilizumab.
CNS-RDD	Involvement of the CNS, enlargement of surrounding lymph nodes, visual changes, pituitary dysfunction and spinal cord dysfunction.	Multiple occupying, meningioma-like lesions in the brain and the spinal cord with obvious homogeneous enhancement and cystic degeneration, and involvement of the endocranium/dura mater.	Infiltration of various types of cells, massive Russell bodies and emperipolesis. S-100 (+), CD68 (+), CD163 (+) and CD1a (+).	Surgery, observation, steroid hormones, radiotherapy, cytotoxic drugs, rituximab and immunosuppressants.

*CNS* Central nervous system, *LyG* Lymphomatoid granulomatosis, *CT* Computed tomography, *ECD* Erdheim-Chester disease, *T-LYP* T-cell lymphoma, *IgG4-RD* IgG4-related disease, *RDD* Rosai–Dorfman disease

Chavez et al. [22] reported a median survival of 23 months in LyG patients. Katzenstein et al. [25] conducted a 3-year follow-up, detecting an overall mortality of 63.5% in LyG patients and 86.0% in CNS-LyG patients, and a good prognosis in 64% of patients with primary CNS-LyG. In the present case report, LyG lesions subsided after medication with steroid hormones for three months and IFN-α for 6 months. She was in a good state at 18 months of follow-up, and a regular, long-term follow-up visit is still needed.

## Data Availability

The datasets used and/or analysed during the current study are available from the corresponding author on reasonable request.

## Abbreviations

LyG: Lymphomatoid granulomatosis
CNS: Central nervous system
CT: Computer tomography
MRI: Magnetic resonance imaging
IFN-α: Immunohistochemistry
EBV: Epstein-Barr virus
PLG: Pulmonary lymphomatoid granulomatosis
^1^H-MRS: Proton magnetic resonance spectroscopy
PSL: Prednisolone
SUV: Standard uptake value
EBER: Epstein-Barr encoding region
CIDP: Chronic inflammatory demyelinating polyradiculoneuropathy
PCNSL: Primary central nervous system lymphoma
LCH: Langerhans cell histiocytosis
IVIG: Intravenous immunoglobulin
NCI: National Cancer Institute

## References

[1] Liebow AA, Carrington CR, Friedman PJ (1972). Lymphomatoid granulomatosis. Hum Pathol.

[2] Carbone A, Gloghini A, Dotti G (2008). EBV-associated lymphoproliferative disorders: classification and treatment. Oncologist.

[3] Sigamani E, Chandramohan J, Nair S, Chacko G, Thomas M, Mathew LG (2018). Lymphomatoid granulomatosis: a case series from South India. Indian J Pathol Microbiol.

[4] Roschewski M, Wilson WH (2012). Lymphomatoid granulomatosis. Cancer J.

[5] Moreno-Estébanez A, González-Pinto T, Agirre-Beitia G, González LM (2021). Primary lymphomatoid granulomatosis of the central nervous system: a diagnostic challenge. Neurologia (Engl Ed).

[6] Song JY, Pittaluga S, Dunleavy K, Grant N, White T, Jiang L (2015). Lymphomatoid granulomatosis–a single institute experience: pathologic findings and clinical correlations. Am J Surg Pathol.

[7] Heslop HE. Biology and treatment of Epstein-Barr virus-associated non-hodgkin lymphomas. Hematol Am Soc Hematol Educ Program. 2005:260–6. https://pubmed.ncbi.nlm.nih.gov/16304390/. 10.1182/asheducation-2005.1.260 16304390

[8] Sabattini E, Bacci F, Sagramoso C, Pileri SA (2010). WHO classification of tumours of haematopoietic and lymphoid tissues in 2008: an overview. Pathologica.

[9] Ishiura H, Morikawa M, Hamada M, Watanabe T, Kako S, Chiba S (2008). Lymphomatoid granulomatosis involving central nervous system successfully treated with rituximab alone. Arch Neurol.

[10] Sheehy N, Bird B, O’Briain DS, Daly P, Wilson G (2004). Synchronous regression and progression of pulmonary nodules on chest CT in untreated lymphomatoid granulomatosis. Clin Radiol.

[11] Lee JS, Tuder R, Lynch DA (2000). Lymphomatoid granulomatosis: radiologic features and pathologic correlations. AJR Am J Roentgenol.

[12] Pittaluga S, Wilson WH, Jaffe ES (2008). WHO classification of tumors of Haematopietic and lymphoidtissues.

[13] Patsalides AD, Atac G, Hedge U, Janik J, Grant N, Jaffe ES (2005). Lymphomatoid granulomatosis: abnormalities of the brain at MR imaging. Radiology.

[14] Kiryu S, Okubo T, Takeuchi K, Inoue Y, Endo T, Odawara T (2006). Magnetic resonance imaging and diffusion tensor analysis of lymphomatoid granulomatosis of the brain. Acta Radiol.

[15] Patil AK, Alexander M, Nair B, Chacko G, Mani S, Sudhakar S (2015). Clinical, imaging and histopathological features of isolated CNS lymphomatoid granulomatosis. Indian J Radiol Imaging.

[16] He C, Wang Y, Zhang L, Lu C, Ge W, Zhang Q (2019). Isolated lymphomatoid granulomatosis of the central nervous system: a case report and literature review. Neuropathology.

[17] Gaha M, Souillard-Scemama R, Miquel C, Godon-Hardy S, Naggara O, Meder JF (2013). MR imaging of the brain and spinal cord in lymphomatoid granulomatosis: a case report and review of the literature. J Neuroradiol.

[18] Lucantoni C, De Bonis P, Doglietto F, Esposito G, Larocca LM, Mangiola A (2009). Primary cerebral lymphomatoid granulomatosis: report of four cases and literature review. J Neurooncol.

[19] Harting I, Hartmann M, Jost G, Sommer C, Ahmadi R, Heiland S (2003). Differentiating primary central nervous system lymphoma from glioma in humans using localised proton magnetic resonance spectroscopy. Neurosci Lett.

[20] Schalk E, Krogel C, Scheinpflug K, Mohren M (2009). Lymphomatoid granulomatosis in a patient with rheumatoid arthritis receiving methotrexate: successful treatment with the anti-CD20 antibody mabthera. Onkologie.

[21] Melani C, Jaffe ES, Wilson WH (2020). Pathobiology and treatment of lymphomatoid granulomatosis, a rare EBV-driven disorder. Blood.

[22] Chavez JC, Sandoval-Sus J, Horna P, Dalia S, Bello C, Chevernick P (2016). Lymphomatoid granulomatosis: a single Institution experience and review of the literature. Clin Lymphoma Myeloma Leuk.

[23] Johnston A, Coyle L, Nevell D (2006). Prolonged remission of refractory lymphomatoid granulomatosis after autologous hemopoietic stem cell transplantation with post-transplantation maintenance interferon. Leuk Lymphoma.

[24] Siegloch K, Schmitz N, Wu HS, Friedrichs B, van Imhoff GW, Montoto S (2013). Hematopoietic stem cell transplantation in patients with lymphomatoid granulomatosis: a european group for blood and marrow transplantation report. Biol Blood Marrow Transplant.

[25] Katzenstein AL, Doxtader E, Narendra S (2010). Lymphomatoid granulomatosis: insights gained over 4 decades. Am J Surg Pathol.

